# Next-Generation Tumor Targeting with Genetically Engineered Cell Membrane-Coated Nanoparticles

**DOI:** 10.34133/bdr.0055

**Published:** 2024-10-25

**Authors:** Quazi T. H. Shubhra, Xiaojun Cai, Qiang Cai

**Affiliations:** ^1^ Institute of Chemistry, University of Silesia in Katowice, 40-006 Katowice, Poland.; ^2^ Department of Neurosurgery, Renmin Hospital of Wuhan University, Wuhan 430060, China.; ^3^ School and Hospital of Stomatology, Wenzhou Medical University, Wenzhou 325027, China.

A recent study by Krishnan et al. [1] has made substantial progress in the field of nanomedicine by advancing cell membrane (CM)-coated nanoparticle (CNP) technology through the integration of genetic engineering. This innovative approach harnesses CM coatings to achieve highly effective tumor targeting, thereby enhancing the precision and efficacy of drug delivery systems.

Cancer remains a major obstacle in modern medicine due to its ability to evade the immune system and metastasize, complicating treatment [2,3]. Traditional therapies often suffer from limited specificity and severe side effects, prompting the search for more targeted and biocompatible solutions. CNPs have emerged as a promising strategy for precise tumor targeting. These nanoparticles (NPs), derived from cells such as red blood cells, macrophages, and cancer cells, mimic natural cell surfaces, allowing them to evade immune detection and integrate smoothly into biological systems. This biomimicry provides superior benefits compared to conventional NP coatings like polyethylene glycol, which mainly extend circulation time but lack the same level of biocompatibility and targeting precision. In cancer therapy, cancer CM (CCM)-coated NPs are particularly noteworthy for their ability to exploit the homing properties of cancer cells, leading to enhanced drug accumulation at tumor sites and improved therapeutic efficacy [4].

Despite the progress in CNP technology, ongoing research aims to enhance their functionality further and expand their therapeutic applications [5]. Genetic engineering and modular functionalization are critical areas of focus, enabling the introduction of novel properties to CNPs. Krishnan et al.’s recent work is an important breakthrough in this field. They have developed a modular approach that employs the SpyCatcher–SpyTag system to enhance the versatility and effectiveness of CNPs for cancer therapy (Figure). This system allows for the precise and efficient attachment of various functional molecules, transforming CNPs into a “plug-and-play” platform. By genetically engineering CMs to express SpyCatcher proteins, these NPs can form robust covalent bonds with SpyTag-modified molecules, allowing for the stable attachment of diverse functionalities. This innovation dramatically improves targeting precision and biointerfacing capabilities, paving the way for next-generation nanomedicine platforms.

**Figure.**
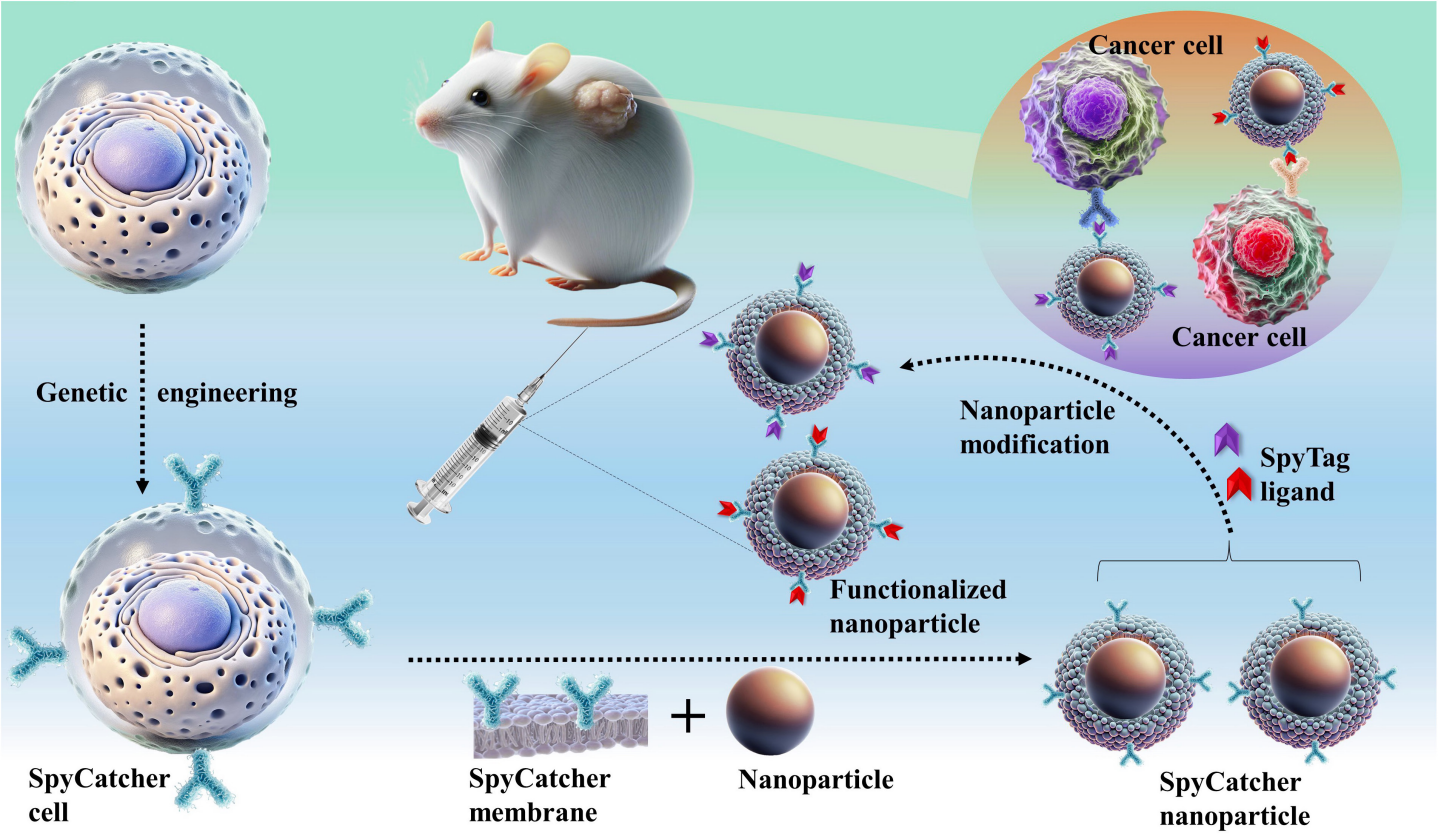
Schematic of the modular approach using the SpyCatcher–SpyTag system for enhancing cell membrane-coated nanoparticles (CNPs) in cancer therapy. The process begins with genetic engineering to produce SpyCatcher-expressing cells, from which SpyCatcher-coated membranes are derived. These membranes are combined with nanoparticle cores to create SpyCatcher-functionalized nanoparticles (SC-NPs). These SC-NPs are then modified with SpyTag-labeled ligands, allowing the attachment of functional molecules. In a tumor-bearing mouse model, the functionalized nanoparticles specifically target cancer cells. This platform enables precise tumor targeting and improved therapeutic efficacy, showing great promise for targeted cancer treatments.

However, the clinical translation of CCM-coated NPs, such as those developed by Krishnan et al., faces regulatory and manufacturing challenges. These NPs may be classified as biologics, complicating approval pathways [4]. Ensuring batch consistency and reducing heterogeneity, similar to extracellular vesicle-based systems, remain key hurdles. To address these issues, developing automated production systems and adopting GMP (good manufacturing practices) protocols for scale-up are essential. Moreover, creating universal cell lines with minimized immunogenic antigens could mitigate immune clearance risks, facilitating the use of nonautologous formulations in clinical settings.

Krishnan et al. developed a stable HEK293 cell line (HEK293-SC) that expresses SpyCatcher proteins on their surface, anchored by a transferrin transmembrane domain and fused with a superfolder green fluorescent protein reporter. This was confirmed using flow cytometry and fluorescence microscopy. To functionalize these CNPs, 4 SpyTag-labeled ligands were used: mKate2 (a fluorescent protein), αmCherry (targeting mCherry), αEGFR (targeting the epidermal growth factor receptor), and αHER2 (targeting human epidermal growth factor receptor 2). These ligands were produced and purified through His-tag affinity chromatography and validated by gel electrophoresis and Western blotting. Functional assays demonstrated that ST-mKate2 bound to HEK293-SC cells in a dose-dependent manner, with fluorescence localized to the cell surface, indicating successful conjugation. Similar specific binding was observed for ST-αmCherry, ST-αEGFR, and ST-αHER2 to their respective receptors, such as ST-αEGFR’s effective binding to epidermal growth factor receptor (EGFR)-positive SKOV3 cells but not to EGFR-negative MDA-MB-453 cells. Plasma membranes from HEK293-SC cells were then coated onto poly(lactic-co-glycolic acid) (PLGA) NP cores to create SpyCatcher-functionalized NPs (SC-NPs), which were optimized at a 1:2 membrane-to-PLGA ratio. PLGA is widely used to prepare nano-drug delivery systems [6–8]. These SC-NPs, approximately 120 nm in size, showed robust stability in isotonic sucrose over 18 days. The formation of mKate2-conjugated NPs was confirmed through size-exclusion chromatography, transmission electron microscopy, and immunogold staining, demonstrating their potential in developing targeted CNPs for cancer nanomedicine and other applications.

The effectiveness of the SpyCatcher–SpyTag system for creating modularly functionalized CNPs for targeted cancer therapy was evaluated through extensive in vitro and in vivo studies. They created targeted formulations such as αmCherry-NPs, αEGFR-NPs, and αHER2-NPs. In vitro, these NPs exhibited precise targeting, with αmCherry-NPs binding specifically to HEK293T-mCherry cells and αEGFR-NPs and αHER2-NPs effectively targeting SKOV3 cells expressing EGFR and HER2. Minimal binding was observed with nonexpressing cells like MDA-MB-453 and MDA-MB-231. Blocking studies confirmed the specificity of these interactions. When loaded with docetaxel (DTX), the targeted NPs (αmCherry-[DTX]NPs, αEGFR-[DTX]NPs, and αHER2-[DTX]NPs) showed potent cytotoxic effects with low IC_50_ values, whereas nontargeted SC-[DTX]NPs and free DTX exhibited minimal cytotoxicity.

In vivo studies with SKOV3 tumor-bearing nude mice showed that αEGFR-NPs and αHER2-NPs accumulated robustly in tumors within an hour after injection and persisted for over 24 h, as confirmed by live and ex vivo imaging. In contrast, nontargeted SC-NPs displayed negligible tumor accumulation. Docetaxel-loaded formulations, αEGFR-[DTX]NPs and αHER2-[DTX]NPs, significantly reduced tumor growth and extended median survival from 29 days (SC-[DTX]NPs) to 63 and 71 days, respectively. Safety assessments revealed no significant changes in body weight or adverse effects, and blood tests in immunocompetent mice showed no differences between treated and control groups, indicating a favorable safety profile.

The modular platform by Krishnan et al. represents a landmark achievement in CNP technology for targeted cancer therapy. The SpyCatcher–SpyTag platform can be adapted to various drug carriers, including lipid-based, metallic, and polymer-based NPs, enabling precise functionalization as outlined in Figure. The fusion of SpyCatcher-expressing membranes with NPs is a crucial step. This versatile approach facilitates the delivery of therapeutic and diagnostic agents, including hybrid CM-coated NPs, enhancing treatment efficacy across diverse cancer types. Future research should explore integrating multimodal therapies into this platform, such as combining chemotherapy with immunotherapy, photothermal therapy, or starvation therapy. This approach could enhance therapeutic efficacy while minimizing drug dosages. Incorporating magnetic NPs into CNPs could also improve targeting precision through magnetic field guidance, enhancing payload localization. Comparative studies between the SpyCatcher–SpyTag system and other dual-targeting strategies, like peptide or aptamer integrations, could reveal their relative strengths in tumor targeting. Expanding the application of this technology beyond oncology is another promising direction. Creating CNPs using macrophage CMs for antibacterial therapies could exploit their natural targeting capabilities and improve treatment against infections [9,10]. Future research should focus on the economic feasibility of producing these advanced systems to facilitate and ensure clinical adoption. Scaling up production and reducing costs will be crucial to making these innovative therapies accessible and practical for widespread use, paving the way for broader integration into medical practice.

In conclusion, Krishnan et al.’s study offers a groundbreaking approach to enhancing CNPs through genetic engineering. This modular platform overcomes traditional CNP limitations, paving the way for next-generation nanomedicines.
